# Nanoparticles as Vectors to Tackle Cancer

**DOI:** 10.3390/biom11111729

**Published:** 2021-11-19

**Authors:** Chengchen Duan, Helen E Townley

**Affiliations:** 1 Nuffield Department of Women’s and Reproductive Health, Oxford University John Radcliffe Hospital, Headington, Oxford OX3 9DU, UK; chengchen.duan@wolfson.ox.ac.uk; 2 Department of Engineering Science, Oxford University, Parks Road, Oxford OX1 3PJ, UK

The aim of this Special Issue, “Nanoparticles for cancer therapy”, was to offer readers a comprehensive and up-to-date insight into the various applications of nanoparticles in cancer treatments. Nanoparticles can vary in size between 1 and 100 nm [1], and may comprise diverse raw materials that are inorganic (e.g., silica, iron oxide, and carbon) or organic (e.g., polymers and liposomes). Nanoparticles can be designed with very large surface areas, resulting in them being widely used in clinical settings to deliver various diagnostic probes and therapeutic reagents [2]. Accumulation in the tumour area may be achieved passively via the enhanced permeability and retention (EPR) effect due to the fenestrated vasculature surrounding tumours [3]. Alternatively, “active” delivery may be achieved by the addition of targeting molecules to the surface, such as antibodies, aptamers, or peptides. Classical methods for evaluating nanoparticle systems include in vitro cell cultures, spheroids (avascular tumour models), and small animal models. However, computer simulation has also come to be another powerful evaluation tool with the help of machine learning (ML). 

Nanoparticles present a promising system for the future of precision and personalised cancer therapy. In this Special Issue, we are pleased to present original research articles and literature reviews which discuss several topical areas within the field, such as synthesis and design, biotoxicity evaluation, and computer modelling of efficacy. One such innovative method to tackle the current obstacles of clinically applying photothermal therapy with nanoparticles was reported by Jeynes et al. [4]. This method was based on Monte Carlo simulations of photon radiative transfer and heat diffusions within tissues in skin cancer. Heat rise simulations were compared and validated with historical experimental data from the relevant literature. The link between heat rise and the concentration of nanorods was also estimated based on the proposed model. The cumulative equivalent minutes at 43 °C (CEM43) model was applied for the evaluation of cancer cell killing efficiency. Photothermal treatment computer modelling provides a powerful tool for thermal dosing characterisation.

A novel treatment for breast cancer was proposed using a chemotherapy–radiotherapy combined system based on chitosan nanoparticles [5]. The system was designed to target genomic instability linked to the spindle assembly checkpoint (SAC), which is a specific feature within breast cancer cells as well as many other types of cancer. The small-molecule inhibitor reversine was delivered into the cancer cells by chitosan nanoparticles. It was found to inhibit Mps1 and Aurora B, which could lead to mitosis, aneuploidy, and cell death with the concomitant use of X-ray irradiation. Assays confirmed that the cancer cell DNA damage and death was triggered by the reversine molecule, rather than the chitosan nanoparticle vector. 

A further study investigated a novel marker for the targeted therapy of glioma [6]. The proton-coupled folate transporter (PCFT) was found to be expressed in Gl261 and A172 glioma cells. The transporter could be used for the selective enhanced uptake of folic acid (FA)-conjugated cytochrome c, containing nanoparticles which led to cell death. Astrocytes which do not express PCFT were unharmed. Thus, the identification of this specific marker provides a new route for treating glioblastomas, since more than a quarter of subtypes overexpressed PCFT and/or folate receptor 1 (FOLR1).

Upconverting nanoparticles (UCNPs) were explored in the article by Wysokińska et al. [7]. The bio-toxicity in macrophages was assessed for one of the most widely applied luminescent probes: NaGdF_4_:Yb^3+^,Er^3+^. Activated macrophages were treated with UCNPs of different sizes but the same zeta potential (c. −11 mV). Incubation with the UCNPs showed a decrease in mitochondrial potential and abnormal pro-apoptotic Bax and anti-apoptotic Bcl-2 regulation, irrespective of the size of the UCNPs. Furthermore, it was discovered that a reversal of the cell acidification that was caused by the UCNPs could avoid biotoxicity. Imaging and treatment were also reviewed in the article by Parodi et al. [8], who considered the application of albumin nanovectors, which was also selected as the Editor’s Choice. As one of the most promising vectors, albumin nanovectors showed tremendous superiority compared with other materials, especially in its biocompatibility, cost efficiency, and flexibility. There are at least seven albumin-specific receptors on cells, including Gp60, BM-40, Gp30, Gp18, and the neonatal Fc receptor for IgG (FcRn). There is also an abundance of reactive sites on albumin, which means that the vectors can be easily functionalised with multiple targeting moieties. Furthermore, albumin-based nanovectors may be administered by intravenous injection or inhalation. 

Another system relied upon reactive oxygen species (ROS) as the agent to cause cancer cell death. α-Tocopheryl succinate (α-TOS)-based polymeric nanoparticles were reported by Sánchez-Rodríguez et al. [9] to be a potential treatment for head and neck squamous cell carcinomas. α-TOS-loaded particles generated the accumulation of reactive oxygen species (ROS) and thereby apoptosis in proliferating endothelial cells, which led to suppressed angiogenesis. Similar results were also validated within the in vitro 3D model. Moreover, the migration of hypopharynx carcinoma cells was shown to be inhibited by α-TOS-loaded NPs through the downregulation of the secretion of matrix metalloproteases 2 and 9 (MMP-2 and MMP-9).

A number of contributions discussed ways in which cancers could be treated by molecules or particles which were activatable. Bertrand et al. [10] discussed the use of the pH-sensitive pro-drug vorinostat, which was loaded into norbornenyl-poly(ethylene oxide) nanoparticles. They also used an innovative method to vectorise histone deacetylase inhibitors (HDACi) with the help of ring-opening metathesis polymerisation (ROMP). The drug could be efficiently released through pH-activated bond cleavage, greatly improving the anti-tumour effect compared with free drugs. An in vivo syngeneic model of mesothelioma in mice showed selective accumulation of the nanoparticles and a decrease in tumour weight of over 80% compared with the control group. The study by Rezaian et al. [11] compared carbon nanotubes (CNTs), fullerene, and graphene oxide (GO) as vectors for the pH-activated delivery and release of doxorubicin (DOX) and paclitaxel (PAX), with the help of N-isopropylacrylamide (PIN). CNTs were found to be the most suitable vectors combined with PIN, due to having the greatest efficiency of adsorption and release as well as superior stability. 

A number of different nanoparticle activation methods were reviewed by White et al. [12]. The ability to control the release of therapeutic compounds results in nanoparticles which can deliver therapy more accurately to the required site of action, and therefore have better efficacy and reduced off-target effects. Six categories of activation methods were discussed: pH, enzymatic, concentration-dependent, ultrasound, magnetic field, and light. Irrespective of whether the activation was intrinsic or extrinsic, the goal was to enable control over the temporal and spatial release of the therapeutic compound in order to enhance specificity. 

In conclusion, this Special Issue explored a number of innovative nanoparticle designs which could lead to new cancer treatments in the future. It is very encouraging to see progress in the understanding of applications as well as new designs and synthesis protocols, along with the evaluation of their efficacy and biotoxicity, both biologically and through computational models. Whilst a tremendous amount of progress has been made at the bench with regard to nanomedicine, it will now be necessary to move forwards with clinical studies. Only then will the potential of these treatments be truly realised. 

## References

[1] ISO-ISO/TS 80004-2:2015-Nanotechnologies—Vocabulary—Part 2: Nano-Objects. https://www.iso.org/standard/54440.html.

[2] Shi J., Kantoff P.W., Wooster R., Farokhzad O.C. (2017). Cancer nanomedicine: Progress, challenges and opportunities. Nat. Rev. Cancer.

[3] Manchun S., Dass C.R., Sriamornsak P. (2012). Targeted therapy for cancer using pH-responsive nanocarrier systems. Life Sci..

[4] Jeynes J.C.G., Wordingham F., Moran L.J., Curnow A., Harries T.J. (2019). Monte Carlo Simulations of Heat Deposition during Photothermal Skin Cancer Therapy Using Nanoparticles. Biomolecules.

[5] Olmos S.P., Torres R.D., Elbakrawy E., Hughes L., McKenna J., Hill M.A., Kadhim M., Noguera P.R., Bolanos-Garcia V.M. (2019). Combinatorial Use of Chitosan Nanoparticles, Reversine, and Ionising Radiation on Breast Cancer Cells Associated with Mitosis Deregulation. Biomolecules.

[6] Kucheryavykh Y.V., Davila J., Ortiz-Rivera J., Inyushin M., Almodovar L., Mayol M., Morales-Cruz M., Cruz-Montañez A., Barcelo-Bovea V., Griebenow K. (2019). Targeted Delivery of Nanoparticulate Cytochrome C into Glioma Cells Through the Proton-Coupled Folate Transporter. Biomolecules.

[7] Wysokińska E., Cichos J., Kowalczyk A., Karbowiak M., Strzadała L., Bednarkiewicz A., Kałas W. (2019). Toxicity Mechanism of Low Doses of NaGdF_4_:Yb^3+^,Er^3+^ Upconverting Nanoparticles in Activated Macrophage Cell Lines. Biomolecules.

[8] Parodi A., Miao J., Soond S.M., Rudzińska M., Zamyatnin A.A. (2019). Albumin Nanovectors in Cancer Therapy and Imaging. Biomolecules.

[9] Sánchez-Rodríguez C., Palao-Suay R., Rodrigáñez L., Aguilar M.R., Martín-Saldaña S., Román J.S., Sanz-Fernández R. (2018). α-Tocopheryl Succinate-Based Polymeric Nanoparticles for the Treatment of Head and Neck Squamous Cell Carcinoma. Biomolecules.

[10] Bertrand P., Blanquart C., Héroguez V. (2019). The ROMP: A Powerful Approach to Synthesize Novel pH-Sensitive Nanoparticles for Tumor Therapy. Biomolecules.

[11] Rezaian M., Maleki R., Dahroud M.D., Alamdari A., Alimohammadi M. (2018). pH-Sensitive Co-Adsorption/Release of Doxorubicin and Paclitaxel by Carbon Nanotube, Fullerene, and Graphene Oxide in Combination with N-isopropylacrylamide: A Molecular Dynamics Study. Biomolecules.

[12] White B.D., Duan C., Townley H.E. (2019). Nanoparticle Activation Methods in Cancer Treatment. Biomolecules.

